# Ethical Considerations in the Use of Artificial Intelligence and Machine Learning in Health Care: A Comprehensive Review

**DOI:** 10.7759/cureus.62443

**Published:** 2024-06-15

**Authors:** Mitul Harishbhai Tilala, Pradeep Kumar Chenchala, Ashok Choppadandi, Jagbir Kaur, Savitha Naguri, Rahul Saoji, Bhanu Devaguptapu

**Affiliations:** 1 Software Engineering, Independent Researcher, Trenton, USA; 2 Software Development, Independent Researcher, Seattle, USA; 3 Data Architecture, Independent Researcher, McKinney, USA; 4 Program Management, Independent Researcher, West Orange, USA; 5 Data Analytics, Independent Researcher, Roswell, USA; 6 Data Analytics, Independent Researcher, Dallas, USA; 7 Data Architecture, Independent Researcher, Rancho Cordova, USA

**Keywords:** professional responsibility, clinical validation, transparency, algorithmic bias, data security, privacy, ethics, healthcare, machine learning, artificial intelligence

## Abstract

Artificial intelligence (AI) and machine learning (ML) technologies are revolutionizing health care by offering unprecedented opportunities to enhance patient care, optimize clinical workflows, and advance medical research. However, the integration of AI and ML into healthcare systems raises significant ethical considerations that must be carefully addressed to ensure responsible and equitable deployment. This comprehensive review explored the multifaceted ethical considerations surrounding the use of AI and ML in health care, including privacy and data security, algorithmic bias, transparency, clinical validation, and professional responsibility. By critically examining these ethical dimensions, stakeholders can navigate the ethical complexities of AI and ML integration in health care, while safeguarding patient welfare and upholding ethical principles. By embracing ethical best practices and fostering collaboration across interdisciplinary teams, the healthcare community can harness the full potential of AI and ML technologies to usher in a new era of personalized data-driven health care that prioritizes patient well-being and equity.

## Introduction and background

Artificial intelligence (AI) and machine learning (ML) technologies represent a paradigm shift in health care, offering unprecedented opportunities to enhance patient care, optimize clinical workflows, and revolutionize medical research [1,2]. The integration of AI and ML into healthcare systems has demonstrated remarkable potential in improving diagnostics, treatment planning, patient monitoring, and drug development. From predictive analytics to personalized medicine, these technologies hold the promise of transforming healthcare delivery and addressing longstanding challenges in the field [3].

The capacity of AI and ML algorithms to analyze vast amounts of complex data, including medical images, genomic sequences, electronic health records (EHRs), and real-time physiological signals, enables healthcare providers to extract valuable insights and make informed clinical decisions [4]. For instance, AI-powered diagnostic tools can accurately detect subtle abnormalities in medical images, leading to earlier detection of diseases, such as cancer, and improved patient outcomes. Similarly, ML algorithms can analyze genetic data to identify individuals at a higher risk of developing certain conditions, facilitating targeted interventions and preventive strategies [5].

Moreover, AI and ML have the potential to streamline healthcare operations and improve resource allocations. These technologies can enhance efficiency, reduce costs, and alleviate healthcare workforce shortages by automating administrative tasks, optimizing hospital workflows, and predicting patient admissions and discharges, these technologies can enhance efficiency, reduce costs, and alleviate healthcare workforce shortages [6]. Additionally, AI-driven predictive models can help healthcare organizations anticipate disease outbreaks, allocate resources during public health emergencies, and optimize treatment protocols based on real-time data analysis [7].

Despite their transformative potential, the integration of AI and ML into healthcare systems raises significant ethical considerations that must be carefully addressed to ensure responsible and equitable deployment. The complex interplay between technological innovation, clinical practice, and ethical principles necessitates a thorough examination of the ethical implications of AI and ML in health care [7].

Ethical considerations permeate every aspect of the development, implementation, and utilization of AI and ML in health care. From safeguarding patient privacy and ensuring data security to mitigating algorithmic biases and promoting transparency, ethical principles serve as the cornerstone of responsible AI adoption in healthcare. Moreover, the ethical implications of AI and ML extend beyond individual patient care to broader societal concerns, such as equity in access to healthcare services, the impact on healthcare disparities, and the redistribution of healthcare responsibilities among stakeholders [8,9].

This narrative review aims to delve into the ethical dimensions of AI and ML in health care, examining the ethical principles that underpin responsible AI deployment, and exploring the ethical dilemmas inherent in the use of these technologies. By critically evaluating the ethical considerations surrounding AI and ML in health care, we can identify potential challenges and opportunities for ethical decision-making, inform policy development, and foster a culture of ethical awareness and accountability among healthcare professionals, policymakers, technology developers, and patients.

## Review

Before delving into the specific ethical considerations pertaining to AI and ML in health care, it is crucial to underscore the foundational ethical principles that have guided healthcare practice. These principles serve as moral compasses for healthcare professionals, shaping their interactions with patients, colleagues, and society. By adhering to these ethical principles, healthcare providers strive to uphold the highest standards of patient care and ensure the well-being of individuals under care. Beneficence is a cornerstone of ethical healthcare practice, encapsulating the moral imperative of acting in the best interests of patients. Healthcare professionals are entrusted with the responsibility to promote the health and welfare of their patients and seek to maximize positive outcomes and alleviate suffering to the greatest extent possible. From prescribing medications to performing surgical interventions, every clinical decision is guided by the overarching goal of beneficence, which aims to improve patients' quality of life and enhance their overall well-being [10,11].

Non-maleficence complements beneficence by emphasizing the obligation to avoid harming patients. Healthcare providers recognize that their actions, no matter how well-intentioned, may carry inherent risks and potential adverse effects. As such, they are bound to exercise caution and prudence in their clinical practice, striving to minimize the likelihood of harm and mitigate any foreseeable risks [11]. Whether it involves conducting thorough risk assessments, obtaining informed consent, or implementing safety protocols, the principle of non-maleficence underscores the importance of prioritizing patient safety [11,12]. Autonomy embodies respect for patients' rights to make informed decisions regarding their health care. Central to the concept of autonomy is the recognition of individuals as rational agents capable of self-determination and personal choice [13]. Healthcare professionals are called on to engage patients in shared decision-making processes, providing them with relevant information, options, and support to enable them to make autonomous choices that align with their values and preferences. Respecting patients' autonomy not only fosters trust and collaboration but also upholds their inherent dignity and autonomy as moral agents [14].

Justice serves as the ethical foundation for fair distribution of healthcare resources and equitable access to high-quality care for all individuals. In an ideal healthcare system, every patient would have equal access to essential services and treatments, regardless of their socioeconomic status, race, ethnicity, or other demographic characteristics [15]. Healthcare professionals recognize their role in addressing disparities in healthcare access and outcomes, advocating for policies and practices that promote health equity and social justice. Whether through advocacy efforts, community outreach programs, or resource allocation decisions, healthcare providers strive to uphold the principle of justice and ensure that no individual is denied access to healthcare services based on unjustifiable grounds [16].

Ethical considerations in AI and ML

Privacy and Data Security

Among the foremost ethical concerns surrounding the utilization of AI and ML in health care are the protection of patient privacy and the safeguarding of sensitive medical data. Healthcare systems amass vast repositories of patient information ranging from EHRs to genomic data and imaging studies. The successful deployment of AI algorithms hinges on access to these data for training and validation purposes. However, this access raises legitimate concerns regarding data privacy and security breaches [17]. In the context of AI and ML, privacy concerns extend beyond traditional data security measures and encompass responsible handling and use of sensitive medical information. Healthcare organizations must ensure that patient data are collected, stored, and processed in compliance with privacy regulations and ethical standards. Unauthorized access to patient data can result in the breach of confidentiality, identity theft, or misuse of sensitive medical information, posing significant risks to patient autonomy and trust in the healthcare system [18].

To address these concerns, stringent data security protocols must be implemented throughout the AI and ML lifecycles, from data acquisition and storage to algorithm development and deployment. Encryption techniques, access controls, and robust authentication mechanisms are essential safeguards for preventing unauthorized access and mitigating the risk of data breaches. Moreover, healthcare organizations must prioritize data minimization and anonymization strategies to limit the exposure to sensitive patient information and mitigate the privacy risks associated with AI and ML applications [19]. Furthermore, adherence to privacy regulations such as the Health Insurance Portability and Accountability Act (HIPAA) in the United States [20], the General Data Protection Regulation (GDPR) in the European Union [21], and similar laws and regulations worldwide is imperative to safeguard patient confidentiality and ensure compliance with legal and ethical standards. These regulations outline specific requirements for the collection, use, and disclosure of protected health information, imposing strict penalties for non-compliance and reinforcing patients' rights to privacy and data protection.

In addition to technical safeguards and regulatory compliance, ethical considerations regarding privacy and data security in AI and ML necessitate a broader commitment to transparency, accountability, and ethical oversight. Healthcare organizations must be transparent to patients about the use of their data for AI and ML applications, providing clear information about data handling practices, potential risks, and privacy safeguards [22,23]. Moreover, mechanisms for ethical review and oversight should be established to evaluate the ethical implications of AI and ML projects, assess risks to patient privacy, and ensure that ethical standards are upheld throughout the development and deployment processes [22,23]. By addressing privacy and data security concerns proactively and transparently, healthcare organizations can build trust with patients, mitigate ethical risks associated with AI and ML applications, and harness the full potential of these technologies to improve patient care and advance medical research, while safeguarding patient privacy and autonomy.

Bias and Fairness

Another critical ethical consideration in the realms of AI and ML in healthcare is the pervasive issue of algorithmic bias and its implications for fairness and equity in healthcare delivery. AI and ML algorithms are susceptible to bias, which can manifest in various forms, including racial, sex, and socioeconomic biases. Biases may stem from skewed training datasets that fail to adequately represent diverse patient populations or from algorithmic design flaws that perpetuate discriminatory outcomes [22,24]. In the context of health care, algorithmic bias poses significant ethical challenges as it can lead to disparities in diagnosis, treatment, and outcomes among different patient groups. For example, a diagnostic algorithm trained predominantly on data from a specific demographic group may be less accurate when applied to individuals from underrepresented populations, leading to misdiagnoses or delayed treatment. Similarly, predictive models that rely on socioeconomic factors may inadvertently perpetuate disparities in access to care or resource allocation, further exacerbating health inequalities [25,26].

If left unaddressed, algorithmic bias can undermine the principles of fairness, justice, and equity in health care, perpetuating systemic discrimination and eroding trust in the healthcare system [27]. Moreover, bias in AI and ML algorithms not only harms individual patients but also has broader societal implications, reinforcing existing disparities and hindering efforts to achieve health equity [28].

To mitigate the bias in AI and ML algorithms, concerted efforts are required across multiple fronts, including data collection, algorithm development, and model evaluation. First, healthcare organizations must prioritize the diversity and representativeness of training data, ensuring that datasets are inclusive of diverse patient populations and account for demographic, socioeconomic, and cultural factors. This may involve actively seeking and incorporating data from underrepresented groups, employing data augmentation techniques to address imbalances, and collaborating with diverse stakeholders to ensure the inclusivity of data collection efforts [28,29]. Furthermore, algorithm developers must employ algorithmic fairness techniques to detect and mitigate biases in the AI and ML models. These techniques may include fairness-aware training algorithms, which aim to optimize models for fairness metrics, such as demographic parity or equalized odds, and post-processing methods, which adjust model outputs to reduce disparate impacts on different groups. Additionally, transparent and interpretable ML techniques can facilitate the identification and explanation of bias in algorithmic decision-making, enabling stakeholders to understand how biases arise and develop strategies to address them effectively [30].

Regular audits and evaluations of AI and ML algorithms are also essential to monitor bias-induced disparities in healthcare outcomes and to ensure that algorithms remain fair and equitable over time. Healthcare organizations should establish mechanisms for ongoing monitoring and evaluation of AI and ML applications involving multidisciplinary teams of clinicians, data scientists, ethicists, and community representatives to assess algorithm performance, identify bias-related risks, and implement corrective actions as needed [28,31]. By addressing bias and promoting fairness in AI and ML algorithms, healthcare organizations can uphold the ethical principles of justice, equity, and non-discrimination, ensuring that AI-driven healthcare technologies benefit all patients regardless of race, gender, socioeconomic status, or other demographic factors [32]. Moreover, by fostering a culture of inclusivity, transparency, and accountability, stakeholders can build trust with patients and communities, mitigate the risk of bias-induced harm, and harness the full potential of AI and ML to advance health equity and improve patient outcomes [33].

Transparency and Explainability

The opacity of some AI and ML models poses significant challenges regarding transparency and explainability, which are essential for fostering trust and accountability in healthcare practices. Healthcare providers and patients alike may struggle to comprehend the underlying mechanisms through which AI algorithms arrive at their clinical predictions or treatment recommendations. This lack of transparency can engender skepticism and erode trust in AI-driven healthcare technologies, potentially hindering their adoption and acceptance in clinical practice [34]. In the context of health care, transparency refers to the openness and accessibility of information regarding the functioning and decision-making processes of AI and ML algorithms. On the other hand, explainability pertains to stakeholders’ ability to understand and interpret algorithmic outputs in a meaningful and comprehensible manner. Both transparency and explainability are critical for ensuring that healthcare providers and patients can trust the recommendations generated by AI and ML models and make informed decisions regarding patient care [35]. To address the challenges of transparency and explainability in AI and ML in health care, concerted efforts are required to enhance the interpretability of algorithmic outputs and provide meaningful explanations for AI-driven recommendations [34]. One approach is to employ techniques for model interpretability, which aims to elucidate the factors contributing to algorithmic predictions and highlight the most influential features or variables. Techniques such as feature importance analysis, partial dependence plots, and local interpretable model-agnostic explanations (LIME) can help healthcare providers understand how AI algorithms arrive at their clinical decisions and identify potential sources of bias or error [36].

Furthermore, healthcare organizations can promote transparency by providing algorithmic transparency reports that document the development, validation, and performance of AI and ML models in a clear and accessible manner. These reports may include information regarding the training data used, algorithmic architecture and parameters, performance metrics, and potential limitations or caveats associated with algorithmic predictions. By providing transparent documentation of AI-driven decision-making processes, healthcare organizations can instill confidence in the reliability and validity of algorithmic outputs and facilitate informed decision-making by healthcare providers and patients [34,37]. In addition to technical approaches, efforts to enhance transparency and explainability in AI and ML in healthcare should also encompass plain language explanations of AI-driven recommendations. Healthcare providers and patients may not have expertise in ML or data science, making it essential to translate algorithmic outputs into terms that are meaningful and relevant to clinical practice. By providing clear and concise explanations of AI-driven recommendations in a language that is accessible to non-experts, healthcare organizations can empower stakeholders to critically evaluate algorithmic outputs and make informed decisions about patient care [38].

By prioritizing transparency and explainability in AI and ML in health care, stakeholders can foster the trust, accountability, and acceptance of these technologies in clinical practice. By ensuring that healthcare providers and patients understand the rationale behind algorithmic recommendations and the limitations of AI-driven decision-making, healthcare organizations can promote the ethical and responsible use of AI and ML in health care, ultimately improving patient outcomes and advancing the delivery of personalized, evidence-based care [34,39].

Clinical Validation and Regulation

The rapid proliferation of AI and ML technologies in healthcare necessitates robust clinical validation studies and stringent regulatory oversight to ensure patient safety and efficacy [1]. Unlike traditional medical interventions that undergo rigorous clinical trials before receiving regulatory approval, AI and ML algorithms pose unique challenges in terms of validation and regulation. The iterative nature of algorithmic development coupled with the dynamic nature of healthcare data complicates the assessment of the clinical validity and reliability of AI-driven technologies [40]. Clinical validation is a critical step in the deployment of AI and ML algorithms in health care to ensure that these technologies deliver accurate, reliable, and clinically relevant results. However, traditional validation methodologies may not be well-suited to the dynamic and evolving nature of AI and ML algorithms. Unlike pharmaceutical drugs or medical devices that undergo extensive preclinical and clinical testing, AI algorithms may undergo continuous updates and improvements, requiring ongoing validation to ensure that their performance remains consistent over time [41].

To address these challenges, there is a pressing need for standardized frameworks and methodologies to validate AI and ML algorithms in healthcare settings. These frameworks should encompass rigorous testing protocols, standardized performance metrics, and guidelines for data collection, model development, and validation study design. Moreover, validation studies should involve diverse patient populations and real-world clinical scenarios to ensure the generalizability and scalability of AI-driven technologies across different healthcare settings and patient demographics [42]. Regulatory oversight is another crucial aspect for ensuring the safety and efficacy of AI and ML algorithms in health care. Regulatory bodies, such as the United States Food and Drug Administration (FDA), play a pivotal role in evaluating and approving AI-based medical devices, thereby safeguarding patient welfare and ensuring adherence to ethical standards. The FDA's regulatory framework for medical devices provides guidance on the classification, premarket review, and post-market surveillance of AI-based products, outlining the requirements for clinical validation, risk assessment, and labeling of these technologies [43].

In addition to regulatory oversight, industry-wide collaboration and knowledge sharing are essential to advance the validation and regulation of AI and ML in health care. Multistakeholder partnerships involving healthcare providers, technology developers, regulatory agencies, and patient advocacy groups can facilitate the development of consensus standards and best practices for validating and regulating AI-driven technologies. These collaborations can also foster transparency and accountability in the deployment of AI and ML algorithms, promoting public trust and confidence in these technologies [44,45]. Overall, clinical validation and regulation are essential components of responsible AI adoption in health care, ensuring that AI and ML algorithms meet rigorous safety, efficacy, and reliability standards. By establishing standardized validation frameworks, enhancing regulatory oversight, and promoting industry-wide collaboration, stakeholders can mitigate risks, maximize benefits, and improve patient outcomes by integrating AI and ML technologies into clinical practice.

Professional Responsibility and Accountability

Healthcare professionals bear a profound ethical responsibility to critically evaluate and integrate AI and ML technologies into clinical practice while upholding the highest standards of patient care and safety. As stewards of patient well-being, healthcare professionals must navigate the complex landscape of AI and ML in health care with diligence, integrity, and commitment to ethical principles [46]. First, healthcare professionals are tasked with exercising vigilance in assessing the validity and reliability of AI-driven recommendations. Although AI and ML technologies hold promise for improving diagnostic accuracy, treatment planning, and patient outcomes, they are not infallible [3].

In clinical practice, AI algorithms can streamline medical analysis and decision-making by processing large volumes of data quickly and identifying patterns that may not be immediately apparent to human clinicians. However, ensuring the validity of each recommendation from an AI algorithm does require clinicians to perform some of the same mental tasks they would without the AI, such as verifying the accuracy and relevance of the data and interpreting results within the context of individual patient circumstances. The benefit of AI in this context lies in its ability to handle routine, time-consuming tasks, thus allowing clinicians to focus more on complex decision-making processes that require human insight and empathy. AI can provide a second opinion, highlight potential diagnoses, suggest treatment options, and monitor patient data continuously, thereby augmenting the clinician's capabilities rather than replacing them. By reducing the cognitive load on healthcare professionals, AI can enhance efficiency, reduce burnout, and potentially improve patient outcomes, as long as it is used as a supportive tool rather than a standalone decision-maker. Healthcare providers must approach AI-generated insights with a critical eye, recognizing the limitations and uncertainties inherent in algorithmic decision-making. This includes understanding the underlying assumptions, biases, and potential sources of error associated with AI and ML algorithms as well as considering alternative explanations and clinical judgment in the interpretation of algorithmic outputs [47].

Moreover, healthcare professionals must recognize and mitigate the risks associated with the use of AI and ML technologies in clinical practice. This may involve questioning the appropriateness of algorithmic recommendations, seeking additional clinical evidence, or consultation when uncertainty arises, and advocating for patient interests in the face of conflicting priorities or pressures. In cases where AI-driven recommendations may lead to adverse outcomes or harm to patients, healthcare providers must be prepared to intervene decisively to safeguard patient well-being, even if it means overriding or disregarding algorithmic suggestions [2,3]. In addition to individual professional responsibility, clear lines of accountability must be delineated to ensure that all stakeholders involved in the development, deployment, and regulation of AI and ML technologies in health care are held accountable for their actions and decisions. Developers, healthcare organizations, and regulatory bodies each have a role to play in promoting the responsible use of AI and ML and mitigating potential risks to patient safety and welfare. This includes ensuring transparency in algorithmic development and deployment processes, adhering to ethical and regulatory standards for data privacy and security, and establishing mechanisms for reporting and addressing adverse events or unintended consequences arising from the use of AI and ML in health care. The practical integration of AI and ML technologies into clinical practice requires a multifaceted approach that addresses several key considerations. Clinicians should receive comprehensive training on how AI functions, its limitations, and potential biases. This training should be incorporated into medical education programs and continuing education for practicing clinicians to enable them to make informed decisions about AI use in patient care. Healthcare professionals must have access to transparent and interpretable AI systems, understand how AI algorithms make decisions, and be able to interpret their outputs, fostering trust and facilitating error detection. AI systems in health care should undergo continual evaluation and improvement, with clinicians involved in monitoring performance and providing feedback for optimization [48].

By fostering a culture of accountability and transparency, healthcare stakeholders can mitigate risks and maximize the benefits of AI and ML integration, while upholding ethical principles and ensuring patient-centered care. This requires collaboration, communication, and a shared commitment to ethical values across healthcare ecosystems. Ultimately, the integration of AI and ML technologies into clinical practice requires a nuanced understanding of the ethical considerations at play and steadfast dedication to upholding professional standards and patient welfare in the pursuit of improved healthcare outcomes [39].

## Conclusions

In conclusion, the integration of AI and ML technologies holds immense promise for transforming healthcare delivery, improving patient outcomes, and advancing medical research. However, this transformative potential is accompanied by myriad ethical considerations that require careful consideration and proactive mitigation strategies. By addressing issues such as data privacy and security, algorithmic bias, transparency, clinical validation, and professional responsibility, healthcare stakeholders can navigate the ethical complexities surrounding AI and ML integration in health care, while safeguarding patient welfare and upholding the principles of beneficence, non-maleficence, autonomy, and justice. By embracing ethical best practices and fostering collaboration across interdisciplinary teams, the healthcare community can harness the full potential of AI and ML technologies to usher in a new era of personalized data-driven health care that prioritizes patient well-being and equity.
